# Aqueous Extract of *Davallia mariesii* Attenuates 6-Hydroxydopamine-Induced Oxidative Damage and Apoptosis in B35 Cells Through Inhibition of Caspase Cascade and Activation of PI3K/AKT/GSK-3β Pathway

**DOI:** 10.3390/nu10101449

**Published:** 2018-10-06

**Authors:** Chi-Rei Wu, Hung-Chi Chang, Yih-Dih Cheng, Wan-Cheng Lan, Shu-Er Yang, Hui Ching

**Affiliations:** 1 The Department of Chinese Pharmaceutical Sciences and Chinese Medicine Resources, China Medical University, Taichung 40402, Taiwan; magic1986713@hotmail.com; 2 Department of Golden-Ager Industry Management, College of Management, Chaoyang University of Technology, Taichung 41394, Taiwan; 3 Department of Pharmacy, China Medical University Hospital, Taichung 40402, Taiwan; M99553@mail.cmuh.org.tw; 4 Department of Beauty Science and Graduate, Institute of Beauty Science Technology, Chienkuo Technology University, Changhua City 500, Taiwan; jessica@ctu.edu.tw; 5 Department of Pharmacy, Taichung Hospital, Ministry of Health and Welfare, Taichung City 403, Taiwan; taic73047@gmail.com

**Keywords:** *Davallia mariesii*, 6-hydroxydopamine, free radical scavenging capacity, intracellular antioxidant defenses, caspase cascade, PI3K/AKT/GSK-3β pathway

## Abstract

The medicinal ferns of Polydiaceae and Davalliaceae species are called “Gusuibu” by Chinese physicians and used as antiaging dietary medicines. Our previous report revealed that *Drynaria fortunei* (Polydiaceae) protected against 6-hydroxydopamine (6-OHDA)-induced oxidative damage via the PI3K/AKT pathway in B35 neuroblastoma cells. The present study compares the antioxidant phytoconstituent contents and radical scavenging capacities of five Davalliaceae species. The further aim was to clarify the protective mechanism of *Davallia mariesii* (DM) against 6-OHDA-induced oxidative damage and apoptosis in B35 cells. The results show that *Araiostegia perdurans* (AP) and DM extracts have better radical scavenging capacities against 1,1-diphenyl-2-picryhydrazyl (DPPH) and reactive oxygen species (ROS) than other Davalliaceae species. However, only DM extract inhibited 6-OHDA autoxidation under cell-free systems and increased cell viability, compared to B35 cells solely exposed to 6-OHDA. DM extract decreased apoptosis and restored mitochondrial expression in 6-OHDA-treated B35 cells. Additional data indicated that DM extract decreased intracellular ROS and nitric oxide levels generated by 6-OHDA exposure. DM extract also restored glutathione (GSH) levels and the activities of glutathione peroxidase and reductase, and then decreased the elevated malondialdehyde (MDA) levels. Finally, DM extract regulated the protein expression of the caspase cascade and PI3K/AKT/GSK-3β pathways. These results suggest that the protective mechanism of DM extract against 6-OHDA-induced oxidative damage and apoptosis might be related to its radical scavenging capacity, maintaining the mitochondrial function to inhibit the Bcl-2/caspase cascade pathway and activating intracellular antioxidant defenses (GSH recycling, HO-1 and NQO-1) by modulating the activation of the PI3K/AKT/GSK-3β pathway.

## 1. Introduction

Parkinson’s disease (PD) is one of the most common neurodegenerative diseases, with onset at a mean age of 55, characterized by resting tremor, rigidity, bradykinesia and postural instability [1,2,3]. The etiology of PD includes a number of potential factors, such as age, genetic aberrations, or environmentally-derived and endogenous neurotoxins [1]. The environmental exposures or inherited mutation in metabolic pathways might cause the production of toxic substances, such as reactive oxygen species (ROS), from endogenous dopamine or environmentally-derived neurotoxins [3,4,5]. Overproduction of ROS leads to mitochondrial dysfunction and abnormal processing of cellular proteins and triggers the apoptosis of dopaminergic neurons. The pathology revealed the progressive death of dopaminergic neurons in the substantia nigra in the brain of PD patients [1,2,5,6]. 6-hydroxydopamine (6-OHDA), a toxic oxidative dopamine metabolite, is rapidly and non-enzymatically oxidized by molecular oxygen to form *p*-quinone and ROS under physiological conditions [3,7]. Both necrotic and apoptotic mechanisms of cell death occur in response to 6-OHDA toxicology. Several studies have pinpointed the important role of the caspase cascade pathway and the phosphoinositide 3 kinase (PI3K)/AKT cascade pathway in 6-OHDA-induced apoptosis and oxidative stress [8,9,10]. Glycogen synthase kinase 3β (GSK-3β) is also associated with the fate of dopaminergic neurons in PD and 6-OHDA-induced neuron death. GSK-3β may exert its toxicity by inducing apoptosis by the direct activation of intrinsic cascades [10,11,12,13]. Hence, 6-OHDA is a widely used tool for investigating pathogenesis and progression of and drug development for PD.

“Gusuibu” has been used in the treatment of osteoporosis and aging-associated symptoms in traditional Chinese medicine. Accumulating reports have shown that it has antioxidative, anti-inflammatory and osteoprotective effects [14]. In Taiwan herb markets, “Gusuibu” is composed of the medicinal ferns of Polydiaceae or Davalliaceae species. The two medicinal ferns from the Polydiaceae species in Taiwan are *Drynaria fortunei* (Kunze) J. Smith and *Pseudodrynaria coronans* (Wall.) Ching. Our previous report revealed that *D. fortunei* protected from 6-OHDA-induced oxidative damage by activating the PI3K/AKT pathway [15]. As for the Davalliaceae species, *Araiostegia perdurans* (Christ) Copel. (AP), *Davallia formosana* Blume (DF), *Davallia griffithiana* Hook. (DG), *Davallia mariesii* Moore ex Baker (DM) and *Davallia solida* (Forst.) Swartz (DS) have been used as “Gusuibu” for dietary antiaging medicines in Taiwan for many years. Our previous report indicated that they have outstanding antioxidant activities [16]. Recent researchers found that DF possessed anti-osteoporotic and antidiabetic effects [17,18]. However, there is no reported literature about the protective effects of the Davalliaceae species against 6-OHDA-induced oxidative damage. Therefore, the present study compares the antioxidant phytoconstituent contents and radical scavenging capacities of five Davalliaceae species, using microtiter spectrophotometric and spectrofluorimetric methods and high-performance liquid chromatography with photodiode array detector (HPLC-DAD). Then, we wanted to clarify the protective mechanism of DM extract against 6-OHDA-induced oxidative damage and apoptosis in B35 neuroblastoma cells.

## 2. Materials and Methods

### 2.1. Plant Collection and Preparation

Five Davalliaceae ferns (AP, DF, DG, DM and DS) were identified and provided by Hung-Chi Chang of Chaoyang University of Technology in Taiwan. Five Davalliaceae plants were extracted with distilled water by sonication and the resulting extracts were concentrated under reduced pressure to obtain AP, DF, DG, DM, or DS extract [16]. AP, DF, DG, DM, or DS extract was dissolved in distilled water to assess antioxidant phytoconstituents contents and radical scavenging activities.

### 2.2. Chemicals

1,1-diphenyl-2-picryhydrazyl (DPPH), 3-(4,5-dimethylthiazol-2-yl)-2,5-diphenyl tetrazolium (MTT), 4-amino-5-methylamino-2’,7’-difluorofluorescein diacetate (DAF-FM DA), 4’,6-diamidino-2- phenylindole dihydrochloride (DAPI), 6-hydroxy-2,5,7,8-tetramethy-chroman-2-carboxylic acid (trolox), 6-hydroxydopamine bromide (6-OHDA), 2’,7’-dichlorofluorescein diacetate (DCFH-DA), acridine orange (AO), ascorbic acid, (+)-catechin, caffeic acid, dimethyl sulfoxide (DMSO), epicatechin, ferrous sulfate heptahydrate, Folic-Ciocalteu’s phenol reagent (FCP), gallic acid, glutathione (GSH), glutathione peroxidase (GPx), glutathione reductase (GR), hydrogen chloride, malodialdehyde (MDA), mangiferin, phosphate buffered saline (PBS), quercetin, sodium molybdate, sodium nitrate, superoxide dismutase (SOD), thiobarbituric acid (TBA), trichloroacetic acid (TCA), vanillic acid, verbascoside, xanthine, and xanthine oxidase (XO) were obtained from Sigma-Aldrich Chemical Co (St. Louis, MO, USA). Hydrogen peroxide (H_2_O_2_) and all HPLC-grade solvents were acquired from Merck (Darmstadt, Germany). Dulbecco’s modified Eagles medium (DMEM) and fetal bovine serum (FBS) were purchased from Gibco (Grand Island, NY, USA). Double distilled water was used throughout the experiments.

### 2.3. Determination of Antioxidant Phytoconstituents by a Spectrophotometric Reader

The antioxidant phytoconstituent contents in the five Davalliaceae species extracts were assayed using 96-well microtiter spectrophotometric methods, according to our previous report [15]. The total phenolic contents were measured through a redox reaction with FCP reagent and expressed as milligram of catechin equivalents per gram of sample. The total phenylpropanoid contents were determined with Arnow reagent (5% *(w/v)* sodium nitrate and 5% *(w/v)* sodium molybdate) and expressed as milligram of verbascoside equivalents per gram of sample. The flavonol and anthocyanidin contents were measured by switching the absorbance wavelength with different hydrogen chloride concentrations and expressed as milligram of quercetin equivalents per gram of sample.

### 2.4. Determination of Phenolic Compounds by HPLC-DAD

*Davallia mariesii* extract was dissolved in distilled water and then filtered using a 0.22 μm filter. The stock solutions of all standards were prepared in methanol. All standard and sample solutions were injected into 20 μL in triplicate. The Shimadzu VP series HPLC and Class-VP^TM^ chromatography data system were used. All chromatographic operations were carried out at room temperature (RT). A LiChrospher® RP-18e (250 × 4 mm, 5 μm) column (Merck KGaA, Darmstadt, Germany) was used. Chromatographic separation of phenolic compounds, including caffeic acid, catechin, epicatechin, gallic acid, mangiferin, and vanillic acid was carried out using a two-solvent system. Solvent A was 100% methanol, and solvent B was 50 mM NaH_2_PO_4_/10% methanol at pH = 3.23. The analyses were performed by a gradient program. The conditions were as follow: Initial condition of 100% solvent B, 0–10 min changed to 90% solvent B, 10–15 min changed to 85% solvent B, 15–20 min changed to 80% solvent B, 20–25 min changed to 67% solvent B, 25–32 min changed to 60% solvent B, and 32–36 min changed to 100% solvent B. Signals were detected at 280 nm. The used concentrations for the calibration of reference phenolic compounds were between 10 and 150 μg/mL. The chromatographic peaks of the phenolic compounds were confirmed by comparing their retention times and ultraviolet (UV) spectra.

### 2.5. Determination of Radical Scavenging Activity in Vitro

The scavenging capacities of DPPH radical or ROS were determined by spectrophotometric or spectrofluorimetric microplate readers (Bio-Tek, PowerWave X340 or FLX800, Winooski, VT, USA), according to our previous studies [15,19]. The scavenging capacity of the DPPH radical is expressed as (+)-catechin equivalents in milligram per gram of sample. The superoxide anion scavenging activity is expressed as SOD equivalents in unit per milligram of sample. The hydrogen peroxide scavenging activity was expressed as trolox equivalents in μmol per milligram of sample. The hydroxyl radical scavenging activity is expressed as quercetin equivalents in milligram per gram of sample. Ferric reducing antioxidant power (FRAP) assay was performed according to the method followed in our previous report [15]. The results are expressed as the relative trolox equivalents in mmol per gram of sample.

### 2.6. Inhibition of Lipid Peroxidation In Vitro

Rat brain homogenate was used as a source of polyunsaturated fatty acids for determining the extent of lipid peroxidation. The reaction solution, including brain homogenate, ferrous sulfate, ascorbic acid and sample solution, was incubated at 37 °C for 30 min. Then, the thiobarbituric acid reactive substance (TBARS) test was performed by rapidly adding 1.2% *(w/v)* TBA and 10% TCA into the reaction solution. The absorbance of the TBARS supernatant was determined at 532 nm [19].

### 2.7. Inhibition of 6-OHDA Autoxidation In Vitro

The toxicity of 6-OHDA is directly correlated to the rate of autoxidation, which formats the intermediate *p*-quinone. The levels of *p*-quinone were monitored at 490 nm for 3 min at 30-s intervals at 37 °C by a spectrophotometric microplate reader (PowerWaveX, Bio-Tek instruments, Inc., Winooski, VT, USA), according to our previous report [15]. The assay was conducted in a cell-free system under conditions that correspond to cellular 6-OHDA treatments.

### 2.8. Cell Culture and Treatment

Rat B35 neuroblastoma cells (ATCC® CRL-2754™, Manassas, VA, USA) were cultured in DMEM, supplemented with 10% FBS, 100 U/mL penicillin and 100 μg/mL streptomycin in a water-saturated atmosphere with 5% CO_2_ at 37 °C. The cell experiments were performed 24 h after the cells were seeded in 96-well sterile clear-bottom plates (2 × 10^4^ cells/well), 6-well sterile clear-bottom plates (8 × 10^5^ cells/well), or 90-mm sterile clear-bottom dishes (4 × 10^6^ cells/dish). The stock solution of AP or DM extract was dissolved with sterile distilled water and filtered using a 0.22 μM sterile filter. The working solution of AP or DM extract (5–100 μg/mL) was freshly prepared and treated 1 h before the addition of 6-OHDA (50 μM).

### 2.9. Cell Viability

Cell viability was evaluated with the MTT assay, according to our previous report [15]. B35 cells were seeded into 96-well sterile clear-bottom plates. Briefly, the medium was replaced with 500 μg/mL MTT solution 24 h after 6-OHDA exposure. After incubating for 2 h at 37 °C, the cells were washed with PBS and lysed with DMSO. The absorbance of the lysed solution was measured at 570 nm. The experiments were performed in triplicate over four independent experiments. Cell viability is expressed as the percentage relative to untreated cells, which served as the control group (designated 100% viable).

### 2.10. Detection of Morphological Changes and Cell Death

Following 6-OHDA exposure for 24 h in 6-well plates, cell morphology and the stains of cell death and mitochondrial expression were observed using a phase-contrast or fluorescence microscope (Nikon, Tokyo, Japan). DAPI and AO staining were used to detect cell death [20]. MitoTracker Green FM was used to detect viable mitochondrial expression [21] Briefly, control and treated cells were fixed with 4% paraformaldehyde for 30 min at 4 °C, washed with PBS and permeabilized with 0.1% Triton X-100 in PBS for 5 min. Cells were washed with PBS and incubated with DAPI, AO, or MitoTracker Green FM. After washing with PBS, cells were captured with a fluorescence microscope.

### 2.11. Measurement of Intracellular ROS and NO levels

Intracellular ROS levels were measured with a ROS sensitive fluorophore DCFH-DA, according to our previous report [20]. Intracellular nitric oxide (NO) levels were measured with a NO sensitive fluorophore DAF-FM DA [22]. B35 cells were seeded into clear-bottomed black 96-well culture plates. Following incubation with 6-OHDA for 24 h, cells were washed by a Krebs-Hepes buffer (KHB) and treated with DCFH-DA (100 μM) or DAF-FM DA (10 μM). After incubation with the fluorophores for 1 h, cells were washed by the KHB again and added to DMEM without phenol red. Then, the fluorescein intensity was measured at Ex 485/Em 530 by a fluorescent microplate reader. Data are expressed as the percentage relative to untreated cells, which served as the control group (designated 100%).

### 2.12. Measurement of Caspase-3 Activity

Intracellular caspase-3 activities were analyzed with a caspase-3 colorimetric activity assay kit (Millipore, Merck KGaA, Darmstadt, Germany). The assay procedure was performed according to the manufacturer’s protocol. Free *p*-nitroaniline (*p*NA), liberated from degradation of acetyl-Asp-Glu-Val-Asp-*p*-nitroaniline (Ac-DEVD-*p*NA) by activated caspase-3, was measured at an absorbance of 405 nm. Data are expressed as the percentage relative to untreated cells, which served as the control group (designated 100%).

### 2.13. Biochemical Assays

Following incubation with 6-OHDA in 9-cm dishes for 24 h, B35 cells were collected and sonicated on ice. The solution was centrifuged for 15 min at 4 °C, and the supernatant was used to assay antioxidant enzymes. The GSH levels and the activities of GPx and GR were measured as previously reported [19]. GPx and GR activities are expressed as mU per milligram of protein. GSH levels are expressed as pmol per milligram of protein. Lipid peroxidation was measured with TBARS assay [19] and expressed as nmol MDA per milligram of protein.

### 2.14. Western Blotting

Twenty-four hours after 6-OHDA exposure, B35 cells were lysed with ice-cold radioimmunoprecipitation assay (RIPA) lysis buffer. Then, the supernatants were collected after centrifugation, and the protein concentration of the supernatants was quantified using a Bradford protein assay kit (Bio-Rad Laboratories, Inc., Hercules, CA, USA) according to the manufacturer’s guidelines. Samples containing 25 μg protein were separated in 10% polyacrylamide electrophoresis gel and transferred electrophoretically to a polyvinylidene fluoride membrane. The membranes were blocked with 5% nonfat dry milk for one hour at RT and incubated with primary antibodies (Actin, apoptosis-inducing factor (AIF), AKT, B-cell lymphoma 2 (Bcl-2), Caspase-3, GSK-3β, heme oxygenase-1 (HO-1), Nicotinamide adenine dinucleotide 2′-phosphate (NAD(P)H):quinone oxidoreductase (NQO-1), phospho-AKT (threonine 308) (*p*-AKT), phospho-GSK-3β (*p*-GSK-3β), phosphoinositide 3-kinase (PI3-K), or Procaspase-9) overnight at 4 °C. The membranes were washed three times with tris-buffered saline with Tween 20 (TBST) and incubated with an appropriate horseradish peroxidase (HRP) conjugated secondary antibody for one hour at RT. After washing, the membranes were immunostained by chemiluminiscent HRP substrate (Millipore, Burlington, MA, USA). Signals were captured by Las 4000 mini imaging system (Fujifilm, Kanagawa, Japan), and the optical density data were analyzed using MultiGauge v3.0 software (Fujifilm, Kanagawa, Japan).

### 2.15. Statistical Analysis

The data from cell experiments are presented as mean ± SEM. Data were analyzed statistically by one-way analysis of variance (ANOVA), followed by Scheffe’s test, using statistical software SPSS 20.0 for Windows. Probability values less than 0.05 were considered statistically significant.

## 3. Results

### 3.1. Antioxidant Phytoconstituents Contents

Phenolic compounds are the main class of natural antioxidants. The more widespread phenolic compounds include flavonols and anthocyanidins, and the intermediate compounds in the biosynthesis of flavonols and anthocyanidins are phenylpropanoids. Therefore, the present study first compared the contents of the above antioxidant phytoconstituents in five Davalliaceae species extracts using some 96-well microtiter spectrophotometric methods. The contents of the above antioxidant phytoconstituents in the five Davalliaceae species extracts are shown in Table 1. DM extract had the highest total phenolic, phenylpropanoid, and anthocyanidin contents among the five Davalliaceae species extracts. However, the highest content of total flavonols was observed in the DS extract. Then, AP extract had the second highest content of all antioxidant phytoconstituents among the five Davalliaceae species extracts.

### 3.2. Radical Scavenging Capacities

Because the phenolic compound content is closely correlated to the antioxidant activity of a plant, we compared the radical scavenging capacities of the five Davalliaceae species extracts against DPPH radical using a 96-well microtiter spectrophotometric method. The DPPH radical scavenging capacities of the five Davalliaceae species extracts are shown in Table 2. The descending order of DPPH radical scavenging capacities for the five Davalliaceae species extracts is as follows: AP > DM > DS > DF > DG.

Reactive oxygen species (ROS), such as superoxide anions, H_2_O_2_, and hydroxy radicals, are the major free radicals in the human body that can induce oxidation in biomolecules such as lipids. Therefore, we further evaluated the ROS scavenging capacities and lipid peroxidation inhibiting effects of the five Davalliaceae species extracts in vitro. The results are shown in Table 2. AP extract had the highest radical scavenging capacity against superoxide anions and the highest lipid peroxidation inhibiting effect among the five Davalliaceae species extracts. However, the highest scavenging capacity against H_2_O_2_ and hydroxy radicals was the DM extract. Finally, we further evaluated the reducing powers of the five Davalliaceae species extracts using FRAP assay, because the free radical scavenging capacity of plants is closely associated with their reducing power. The descending order of the reducing power of the five Davalliaceae species extracts was as follows: DM > AP > DG > DS > DF (Table 2).

The relationship between the above free radical scavenging capacities and the contents of the above antioxidant phytoconstituents of the five Davalliaceae species extracts is shown in Table 3. The result indicates a significant positive correlation between some radical scavenging capacities and some antioxidant phytoconstituents. Mainly, the total phenolic content was positively and highly correlated with DPPH radical (*r* = 0.89), superoxide anion (*r* = 0.91), and H_2_O_2_ (*r* = 0.996) scavenging capacities. The total phenylpropanoid content was positively and highly correlated with H_2_O_2_ (*r* = 0.95) and hydroxy radical (*r* = 0.94) scavenging capacity. Additionally, the total phenolic content was highly correlated with the total phenylpropanoid content (*r* = 0.95).

### 3.3. Phytoconstituent Profiles of DM by HPLC-DAD

**Figure 1:**
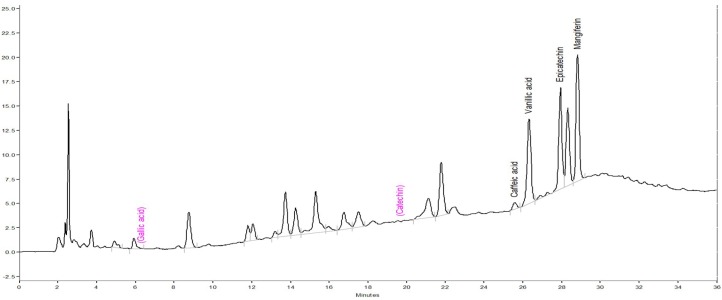
Chromatograms of aqueous extracts of *Davallia mariesii* (DM) with high performance liquid chromatogram (HPLC) at 280 nm.

The phytoconstituent profiles of DM extract were further assayed using HPLC-DAD. The chromatograph is shown in Figure 1. Compared with the retention times and UV spectra of the standard chromatograph, each 10 g of DM extract contained 10.30 ± 0.11 mg epicatechin, 8.34 ± 0.03 mg mangiferin, 5.12 ± 0.09 mg vanillic acid and 3.75 ± 0.01 mg caffeic acid.

### 3.4. Inhibition of 6-OHDA Autoxidation

**Figure 2:**
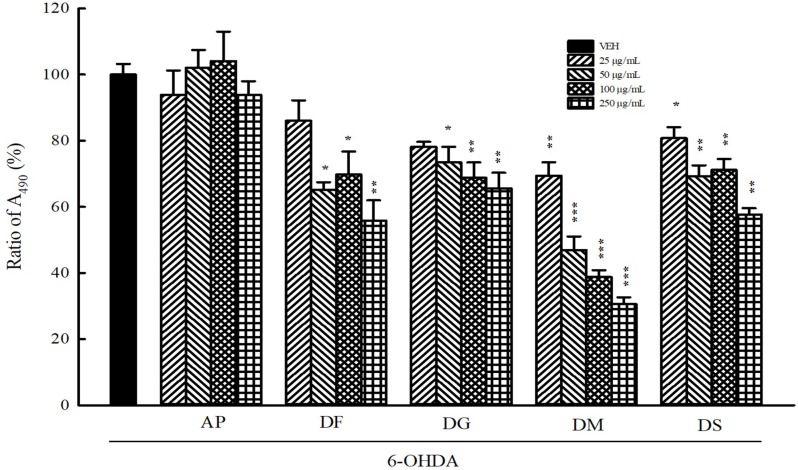
Effect of aqueous extracts of five Davalliaceae species (10–250 μg/mL) on *p*-quinolone production from 6-hydroxydopamine (6-OHDA, 50 μM) autoxidation. Data are expressed as mean ± SEM (*n* = 3). * *p* < 0.05, ** *p* < 0.01, *** *p* < 0.001, compared with VEH group. AP: *Araiostegia perdurans,* DF: *Davallia formosana,* DG: *Davallia griffithiana,* DM: *Davallia mariesii*, DS: *Davallia solida*, VEH: vehicle (distilled water).

Due to 6-OHDA-caused cell toxicology partially through the formation of *p*-quinone from autoxidation, we compared the inhibiting effects of the five Davalliaceae species extracts against 6-OHDA autoxidation by monitoring *p*-quinone production in a cell-free physiological system. DM extract had the highest inhibiting effect against the formation of *p*-quinone from 6-OHDA autoxidation among the five Davalliaceae species extracts (*p* < 0.01, *p* < 0.001, respectively). However, the AP extract did not inhibit the formation of *p*-quinone from 6-OHDA autoxidation at any concentration (*p* > 0.05) (Figure 2).

### 3.5. Protection Against 6-OHDA Toxicology in B35 Cells

**Figure 3:**
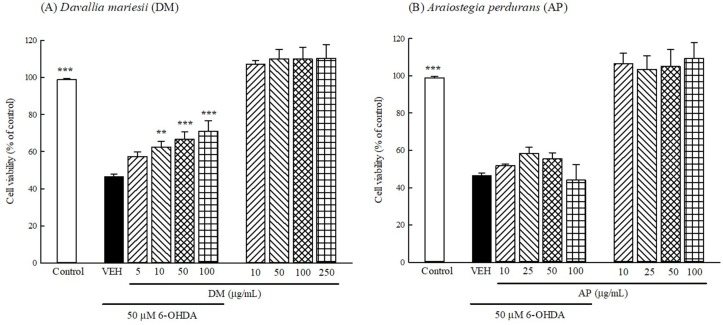
Effect of aqueous extracts of (**A**) *Davallia mariesii* (DM, 5–100 μg/mL) or (**B**) *Araiostegia perdurans* (AP, 5–100 μg/mL) on 6-hydroxydopamine (6-OHDA, 50 μM)-induced toxicology in B35 cells. Data are expressed as mean ± SEM (*n* = 4). AP or DM extract was treated 1 h before the addition of 6-OHDA. Cell viability was measured by 3-(4,5-dimethylthiazol-2-yl)-2,5-diphenyl tetrazolium (MTT) assay. ** *p* < 0.01, *** *p* < 0.001, compared with 6-OHDA/VEH group. VEH: vehicle (DMEM without phenol red).

Next, we evaluated the protective effects of DM or AP extract (10–100 μg/mL) against 6-OHDA-induced toxicology in B35 cells using the MTT assay. When only treated with DM or AP extract (10–250 μg/mL) without 6-OHDA for 24 h, the viability of B35 cells did not significantly change in comparison with that of the control group (Figure 3). After incubation with 50 μM 6-OHDA for 24 h, the cell viability of B35 cells decreased to 46.3% compared with that of control group (*p* < 0.001). DM extract at 10–100 μg/mL increased the cell viability that was decreased by 6-OHDA in a concentration-dependent manner (*p* < 0.01, *p* < 0.001, respectively) (Figure 3A). However, AP at 10–100 μg/mL did not affect the cell viability that has been decreased by 6-OHDA (*p* > 0.05) (Figure 3B).

### 3.6. Cell Morphology and Apoptosis

**Figure 4:**
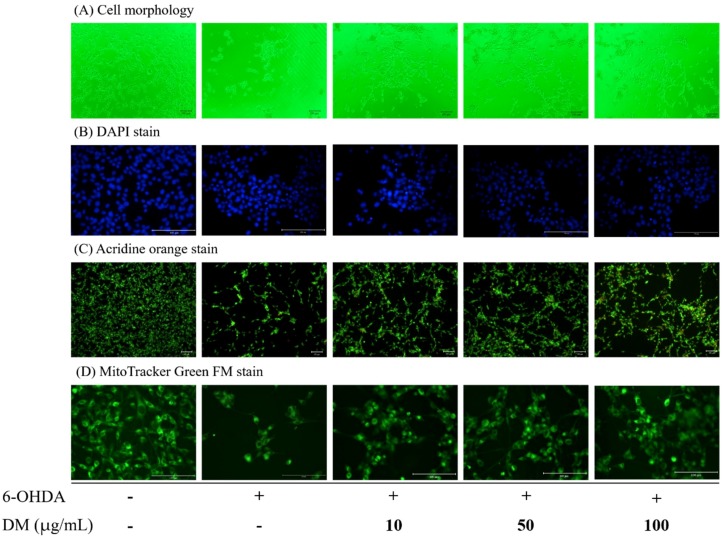
Effect of aqueous extracts of *Davallia mariesii* (DM, 5–100 μg/mL) on 6-hydroxydopamine (6-OHDA, 50 μM)-induced toxicology in B35 cells. (**A**) Cell morphology. (**B**) DAPI stain. (**C**) Acridine orange stain. (**D**) MitoTracker Green FM stain. DM extract was treated 1 h before the addition of 6-OHDA. DAPI: 4’,6-diamidino-2-phenylindole dihydrochloride.

We further observed the morphological alterations of B35 cells using phase-contrast microscopy. Incubation with 50 μM 6-OHDA for 24 h induced cell shrinkage and a decrease in cell numbers. DM extract increased cell numbers in a concentration-dependent manner (Figure 4A). DAPI staining showed the apoptotic cells in 50 μM 6-OHDA treatment for 24 h had increased. DM extract decreased the apoptotic cells. Additionally, AO staining results showed that treatment with 6-OHDA in B35 cells enhanced the apoptotic morphological changes, including chromatin condensation and nuclear fragmentation, and DM extract reversed the apoptotic morphological changes in a concentration-dependent manner (Figure 4B,C). Fluorescence intensity of MitoTracker Green that was used to label mitochondria decreased in B35 cells incubated with 50 μM 6-OHDA for 24 h. DM extract also restored the fluorescence intensity of MitoTracker Green in a concentration-dependent manner (Figure 4D).

### 3.7. Intracellular ROS and NO Production

**Figure 5:**
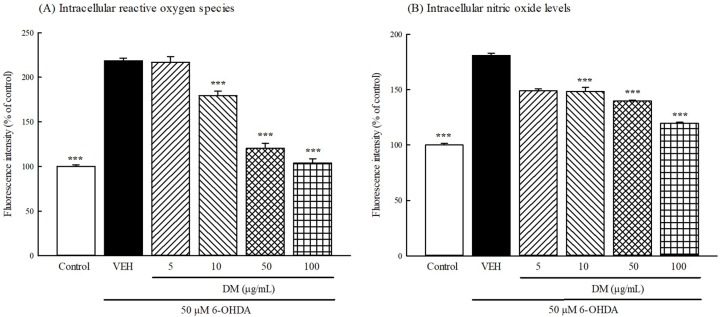
Effect of aqueous extracts of *Davallia mariesii* (DM, 5–100 μg/mL) on 6-hydroxydopamine (6-OHDA, 50 μM)-induced the elevation of intracellular (**A**) reactive oxygen species and (**B**) nitric oxide levels in B35 cells. Data are expressed as mean ± SEM (*n* = 4). DM extract was treated 1 h before the addition of 6-OHDA. Intracellular reactive oxygen species levels were measured by DCFH-DA assay. Intracellular nitric oxide levels were measured by DAF-FM DA assay. *** *p* < 0.001, compared with 6-OHDA/VEH group. VEH: vehicle (DMEM without phenol red).

We investigated whether DM extract countered intracellular ROS and NO production in B35 cells treated with 6-OHDA. As shown in Figure 5A, exposure of B35 cells to 50 μM 6-OHDA for 24 h led to a significant increase in the dichlorofluorescein (DCF) fluorescence signals compared with those of the control group (*p* < 0.001). Pretreatment with DM extract (10–100 μg/mL) significantly decreased DCF fluorescence intensity in a concentration-dependent manner. As shown in Figure 5B, exposure of B35 cells to 50 μM 6-OHDA for 24 h also led to a significant increase in the fluorescence signals of DAF-FM compared with those of the control group (*p* < 0.001). Pretreatment with DM extract (10–100 μg/mL) significantly decreased the fluorescence intensity of DAF-FM in a concentration-dependent manner.

### 3.8. Intracellular Aantioxidant Activities and Oxidative Damage

The GSH recycle system mainly includes GSH, GPx and GR as an important intracellular antioxidant defense system in every cell and tissue. We measured GSH levels and the activities of GPx and GR in B35 cells treated with 50 μM 6-OHDA for 24 h. Incubation with 50 μM 6-OHDA for 24 h decreased intracellular GSH levels in B35 cells (*p* < 0.01), as well as decreased the activities of GPx and GR in B35 cells (*p* < 0.01, *p* < 0.001, respectively) (Table 4). B35 cells pretreated with DM extract at 50–100 μg/mL markedly increased intracellular GSH levels and the activation of GPx and GR compared to cells exposed solely to 6-OHDA (*p* < 0.05, *p* < 0.01, *p* < 0.001, respectively) (Table 4). We also measured lipid peroxidation biomarker MDA levels in B35 cells treated with 50 μM 6-OHDA for 24 h. We found that 6-OHDA (50 μM) caused an increase in the MDA levels in B35 cells (*p* < 0.01) and DM extract at 50–100 μg/mL markedly decreased MDA levels that were elevated by 6-OHDA (*p* < 0.05, *p* < 0.01, respectively) (Table 4).

### 3.9. Caspase Cascade Pathway

**Figure 6:**
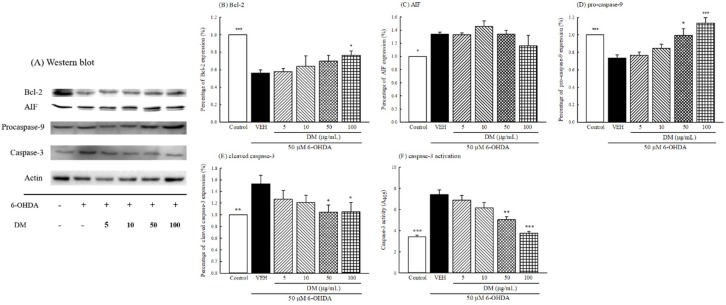
Effect of aqueous extracts of *Davallia mariesii* (DM, 10–100 μg/mL) on 6-hydroxydopamine (6-OHDA, 50 μM)-induced alteration of Bcl-2 and caspase protein expression in B35 cells. (**A**) Protein was determined by immunoblot assay, (**B**) Levels of Bcl-2 expression, (**C**) Levels of apoptosis-inducing factor (AIF) expression, (**D**) Levels of pro-caspase 9 expression, (**E**) Levels of cleaved caspase-3 expression, (**F**) caspase-3 activation. DM extract was treated 1 h before the addition of 6-OHDA. Data are expressed as mean ± SEM (*n* = 3). * *p* < 0.05, ** *p* < 0.01, *** *p* < 0.001, compared with 6-OHDA group.

Immunoblotting was used to determine the effect of DM extract on the protein expression of the caspase cascade pathway in B35 cells treated with 50 μM 6-OHDA. The protein immunoblot assay is shown in Figure 6A. Incubation with 50 μM 6-OHDA for 24 h decreased the levels of Bcl-2 and procaspase-9 proteins (*p* < 0.01, *p* < 0.001) (Figure 6B,D, respectively). Furthermore, 6-OHDA increased the levels of AIF and cleaved caspase-3 proteins in B35 cells (*p* < 0.05, *p* < 0.01) (Figure 6C,E, respectively). B35 cells pretreated with DM extract at 50–100 μg/mL restored the levels of Bcl-2 and procaspase-9 proteins (*p* < 0.05, *p* < 0.01) (Figure 6B,D, respectively), and inhibited the elevated level of cleaved caspase-3 protein (*p* < 0.05) (Figure 6E). However, DM extract did not inhibit the elevated level of AIF protein at any concentration (*p* > 0.05) (Figure 6C).

Furthermore, we investigated whether DM extract countered caspase-3 activation induced by 50 μM 6-OHDA in B35 cells. As shown in Figure 6F, the appearance of *p*NA (caspase-3 activation) was effectively increased by adding 50 μM 6-OHDA. B35 cells pretreated with DM extract at 50–100 μg/mL markedly decreased caspase-3 activation when compared to cells exposed solely to 6-OHDA (*p* < 0.01, *p* < 0.001, respectively) (Figure 6F).

### 3.10. PI3K/AKT/GSK-3β Pathway

**Figure 7:**
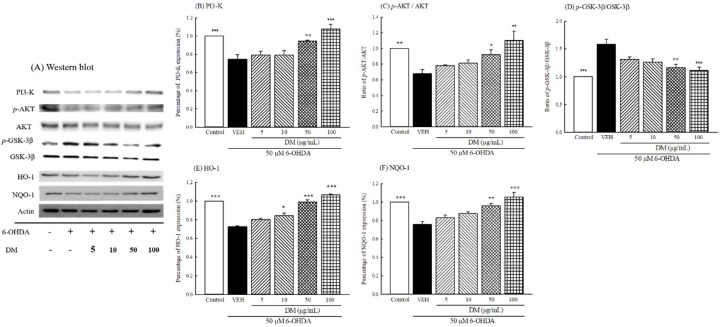
Effect of aqueous extracts of *Davallia mariesii* (DM, 10–100 μg/mL) on 6-hydroxydopamine (6-OHDA, 50 μM)-induced alteration of PI3-K and AKT protein expression in B35 cells. (**A**) Protein was determined by immunoblot assay, (**B**) Levels of PI3-K expression, (**C**) Ratio of p-AKT to AKT, (**D**) Ratio of p-GSK-3β to GSK-3β, (**E**) Levels of HO-1 expression, (**F**) Levels of NQO-1 expression. DM extract was treated 1 h before the addition of 6-OHDA. Data are expressed as mean ± SEM (*n* = 3). * *p* < 0.05, ** *p* < 0.01, *** *p* < 0.001, compared with 6-OHDA group. GSK-3β: glycogen synthase kinase-3β, HO-1: heme oxygenase-1, NQO-1: nicotinamide adenine dinucleotide 2′-phosphate (NAD(P)H):quinone oxidoreductase, *p*-AKT: phospho-AKT (threonine 308), *p*-GSK-3β: phospho-GSK-3β, PI3-K: phosphoinositide 3-kinase.

Immunoblotting was used to further investigate whether DM extract countered the protein expression of the PI3K/AKT/GSK-3β pathway in B35 cells treated with 50 μM 6-OHDA. The protein immunoblot assay is shown in Figure 7A. Incubation with 50 μM 6-OHDA for 24 h decreased the levels of PI3K and the ratio of *p*-AKT (threonine) to AKT (*p* < 0.01) (Figure 7B,C). However, 6-OHDA increased the ratio of *p*-GSK-3β to GSK-3β in B35 cells (*p* < 0.001) (Figure 7D). 6-OHDA further decreased the levels of HO-1 and NQO-1 proteins in B35 cells (*p* < 0.001) (Figure 7E,F). B35 cells pretreated with DM extract at 50–100 μg/mL restored the levels of PI3K, HO-1 and NQO-1 proteins, as well as the ratio of *p*-AKT to AKT (*p* < 0.05, *p* < 0.01, *p* < 0.001, respectively) (Figure 7B–F). In addition, DM extract at 50–100 μg/mL inhibited the elevated ratio of *p*-GSK-3β to GSK-3β (*p* < 0.01, *p* < 0.001) (Figure 7D).

## 4. Discussion

“Gusuibu” is a common traditional Chinese medicine that has been used to prevent aging-associated neurodegenerative disorders, such as PD or Alzheimer’s disease (AD), for centuries [14,15]. The major biochemical pathophysiological processes of these aging-associated neurodegenerative diseases include intracellular oxidative stress, mitochondrial dysfunction, and protein misfolding and dysfunction [1,2,5,6]. Many studies revealed that the overproduction of free radical during oxidative stress plays an important role in the biochemical pathogenesis of PD. Our previous report indicated that the major source of “Gusuibu”—*D. fortunei* (Polydiaceae)—contained enriched total phenolics and flavonoids and had better radical scavenging potency. *D. fortunei* further protected 6-OHDA-induced oxidative damage by activating the PI3K/AKT pathway in B35 neuroblastoma cells [15]. In Chinese medicine materials and the Taiwanese herb market, the medicinal ferns from Davalliaceae species, including AP, DF, DG, DM and DS, are another source of “Gusuibu”. Our present data demonstrated the close relationship between antioxidant phytoconstituent contents (total phenolics) and DPPH radical scavenging capacities of the above five Davalliaceae species, consistent with the findings of our previous reports [15,19,20]. Among the above five Davalliaceae species, AP and DM extracts had the higher total phenolic contents and better DPPH radical scavenging potency.

The biochemical pathogenesis of PD involves the formation of both highly reactive, redox-cycling DA-derived quinones and ROS from excess cytosolic dopamine by monoamine oxidase (MAO) or autoxidation. ROS, mainly including superoxide anions, H_2_O_2_ and hydroxyl radicals, attacked the adjacent biomolecules, such as polyunsaturated fatty acids, proteins and nuclei acids. Our present results showed that there is a close relationship between antioxidant phytoconstituent contents (total phenolics and phenylpropranoids) and ROS radical scavenging capacities of the five Davalliaceae species. DM extract had the best radical scavenging potency against H_2_O_2_ and hydroxy radicals, and the AP extract possessed the best radical scavenging potency against superoxide anions among the five Davalliaceae species. Only DM extract (but not AP extract) at the used concentrations in the ROS-scavenging activity assays inhibited *p*-quinone production from 6-OHDA autoxidation in a concentration-dependent manner. Furthermore, we found that DM extract contains phenolic acids (caffeic acid and vanillic acid), epicatechin and mangiferin from HPLC chromatogram. Some earlier reports confirmed that caffeic acid, epicatechin, mangiferin and vanillic acid possess the antioxidant potency to scavenge various free radicals and inhibit peroxidation [23,24,25,26]. From the above results, we suggest that DM extract inhibited lipid peroxidation in brain homogenate systems mainly by terminating oxidative chain reactions through its better radical scavenging capacity. Then, the effects of DM extract may be based on the higher contents of phenolics and phenylpropranoids, such as epicatechin and mangiferin. However, different phytoconstituent profiles were observed between DM and *Drynaria fortunei* by HPLC-DAD, although both medicinal ferns are major sources of “Gusuibu” in the Taiwanese herb market. This difference might assist with the identification of the “Gusuibu” sources from Polydiaceae or Davalliaceae.

6-hydroxydopamine (6-OHDA), a selective dopaminergic neurotoxin, can lead to some biochemical pathogenesis, such as oxidative damage (excessive ROS and *p*-quinone generation during 6-OHDA autoxidation), mitochondrial dysfunction and apoptosis similar to PD [1,2,5,6]. We found that DM extract, at the used concentrations in the radical scavenging activity assays and 6-OHDA autoxidation, increased cell viability against 6-OHDA in a concentration-dependent manner in B35 cells. It also reversed 6-OHDA-induced morphological changes and mitochondrial expression, and then decreased 6-OHDA-induced apoptosis (chromatin condensation and nuclear fragmentation) in B35 cells. Furthermore, DM extract decreased intracellular oxidative and nitrosative stress by inhibiting ROS and NO production from 6-OHDA in B35 cells.

In addition, intracellular oxidative and nitrosative stress caused lower activities of the intracellular antioxidant defense system, and the lower antioxidant defense increased intracellular ROS and NO production again. The intracellular antioxidant defense system mainly includes several antioxidants and antioxidant enzymes that prevent ROS formation or detoxify ROS. GSH recycling is a major intracellular antioxidant defense system, including GSH and related enzymes, such as GPx and GR. Loss of intracellular GSH levels is often found in the brains of PD patients, as this loss is correlated with the loss of dopaminergic neurons in the substantia nigra and the symptoms of motor dysfunction [1,2,5,27]. Our results are consistent with earlier reports [15,28] that incubation with 6-OHDA for 24 h in B35 cells decreased GSH recycling (including GSH levels and the activities of related enzymes) and increased the levels of oxidative damage marker, MDA. DM extract restored the activities of the GSH recycle antioxidant defense system that had been decreased by 6-OHDA, thereby decreasing the oxidative damage in B35 cells. Therefore, we suggest that DM extract protected against 6-OHDA-induced oxidative damage, mitochondrial dysfunction, and apoptosis in B35 cells, partially via scavenging and inhibiting ROS and *p*-quinone production from 6-OHDA oxidation in B35 cells and upregulating the antioxidant status via intracellular GSH regeneration. Many studies have evidenced that both phytoconstituents of DM extract, mangiferin and epicatechin, possessed neuroprotective and antiapoptotic effects in vitro and in vivo. Mangiferin protected against oxidative stress, mitochondrial dysfunction, and apoptosis caused by 6-OHDA, rotenone, MPTP, amyloid beta oligomers and subarachnoid hemorrhage [28,29,30,31,32]. Epicatechin also had neuroprotective effects against 6-OHDA-induced striatal or amyloid 25-35-induced hippocampal oxidative stress [33,34]. Based on the above results, we suggest that DM is a potential medicinal fern that protects against 6-OHDA-induced oxidative damage, mitochondrial dysfunction and apoptosis.

It was reported that 6-OHDA-induced mitochondrial dysfunction is closely related to the apoptosis of dopaminergic neurons. 6-OHDA-induced mitochondrial dysfunction caused the release of AIF (caspase-independent) and cytochrome c (caspase-dependent) from the mitochondrial intermembrane space into the cytosol. The released AIF migrated into the nucleus and bound to DNA, which triggered DNA destruction and cell apoptosis. The presence of toxic cytochrome c in the cytosol contributes to the activation of caspase-9, followed by the activation of caspase-3, and eventually leads to cell apoptosis [5,6,35]. To elucidate the molecular mechanism of DM extract against 6-OHDA-induced apoptosis in B35 cells, we studied the regulating effects of DM extract on protein expression of the apoptotic pathway and the alteration of caspase-3 activation. The presented results showed that 6-OHDA treatment for 24 h increased AIF protein and decreased Bcl-2 and procaspase-9 protein, and subsequently increased cleaved caspase-3 protein. The caspase-3 activity was markedly increased in B35 cells treated with 6-OHDA. DM extract significantly increased Bcl-2 and procaspase-9 protein, and decreased the activation and protein expression of capase-3. However, there was no difference in the AIF protein between 6-OHDA treatment and DM pretreatment. All these results support that DM extract protects the mitochondria of B35 cells from 6-OHDA-induced apoptosis partially via the Bcl-2/caspase-dependent pathway. Actually, mangiferin protected against oxidative damage and apoptosis caused by rotenone, MPTP amyloid beta oligomers or subarachnoid hemorrhage via modulating the redox balance and inhibiting the mitochondria-dependent caspase cascade [29,30,31,32].

The PI3K/AKT pathway modulates fundamental cellular activities, like neuronal cell proliferation, migration and plasticity. AKT is mainly activated through PI3K, which enhances the formation of phosphatidylinositol (3,4,5)-trisphosphate (PIP3), recruits AKT to the plasma membrane, and induces AKT activation. The PI3K/AKT pathway encourages cell survival and has a cytoprotective function by phosphorylating a variety of enzymes, including pro-apoptotic regulators, detoxifying and antioxidant proteins and transcription factors [10,36]. The related cytoprotective enzymes, such as HO-1 and NQO-1 modulated by the PI3K/AKT pathway, play an important role in neuroprotective functions. HO-1 and NQO-1 proteins strengthen the intrinsic antioxidant potential of cells and detoxify ROS and quinones produced from autoxidation and enzymatic oxidation of dopamine [37,38]. The presented results showed that treatment with 6-OHDA for 24 h decreased PI3K protein and AKT phosphorylation, and subsequently decreased the levels of HO-1 and NQO-1 in B35 cells, consistent with our previous report [15]. DM extract increased PI3K protein and AKT phosphorylation, and restored the levels of HO-1 and NQO-1. GSK-3 is involved in metabolism, gene expression, cell fate determination, proliferation and survival. GSK-3β, one of two GSK-3 isoforms, is known to play critical roles in oxidative stress-induced neuronal apoptosis and the pathogenesis of neurodegenerative diseases. AKT inhibits GSK-3β activities and thereby diminishes apoptosis [12]. We further found DM extract decreased GSK-3β phosphorylation elevated by 6-OHDA in B35 cells. Therefore, we suggest that the DM extract inhibited GSK-3β phosphorylation and restored the protein expression of HO-1 and NQO-1 via activating the PI3K/AKT pathway and inhibiting mitochondria-dependent caspase cascade to counteract oxidative damage, mitochondrial dysfunction and apoptosis caused by 6-OHDA in B35 cells. There are reports about oxidative damage caused by toxins or ischemia-reperfusion, mangiferin and epicatechin protected against cardiac, intestinal, renal, or cerebral damage via activation of the PTEN/PI3K/AKT pathway or Nrf2/HO-1 pathway [39,40,41,42,43]. In fact, mangiferin and epicatechin have a preventative role in aging-associated symptoms and neurodegenerative diseases, as reported by many researchers [26,44,45]. Hence, epicatechin and mangiferin might be active phytoconstituents in the DM extract; however, the contained contents of epicatechin and mangiferin in the used concentrations of DM extract in this present study were lower than those used in other reports [28,29,46]. Further investigation into the synergistic effects of epicatechin and mangiferin still need to be performed because there is a synergistic effect between epicatechin and other phenols [47,48]. Moreover, studies have evidenced that the activation of several signal transduction pathways, such as PI3K/AKT or the mitogen-activated protein kinase (MAPK) pathways, induce Nrf2 nuclear translocation and then upregulate HO-1 to protect dopaminergic neurons against 6-OHDA-induced neurotoxicity. Therefore, the role of mitochondrial enzymes, Bcl-2/Bax and Keap1/Nrf2, in the neuroprotective effects of DM extract and its active phytoconstituents (epicatechin and mangiferin) against 6-OHDA-induced mitochondrial dysfunction and apoptosis must be further investigated.

## 5. Conclusions

**Figure 8:**
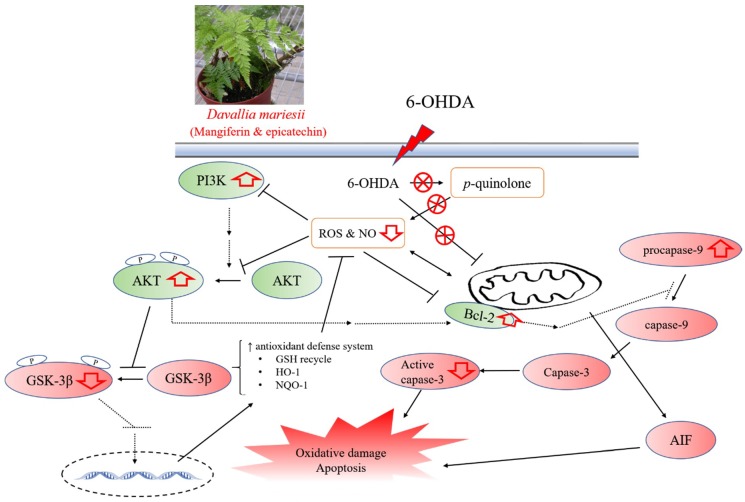
The biological action of *Davallia mariesii* (DM) as a potential antioxidant and protective plant against oxidative stress caused by 6-hydroxydopamine (6-OHDA). Prohibition sign indicates the inhibitory effect of *Davallia mariesii*. AIF: apoptosis-inducing factor, Bcl-2: B-cell lymphoma 2, GSH: glutathione, GSK-3β: glycogen synthase kinase-3β, HO-1: heme oxygenase-1, NO: nitric oxide, NQO-1: nicotinamide adenine dinucleotide 2′-phosphate (NAD(P)H):quinone oxidoreductase, PI3K: phosphoinositide 3 kinase, ROS: reactive oxygen species.

Among five Davalliaceae species extracts, the AP and DM extracts had higher phenolic and phenylpropanoids levels and demonstrated higher radical scavenging capacities. Radical scavenging capacity is closely and positively correlated with the phenolic and phenylpropanoids phytoconstituent contents. Only DF extract inhibited 6-OHDA autoxidation under cell-free conditions and had a protective effect against 6-OHDA-induced oxidative damage and apoptosis in B35 cells. Epicatechin and mangiferin are its major active compounds, because epicatechin and mangiferin can protect against oxidative damage and apoptosis caused by 6-OHDA and amyloid β peptide [28,29,31,33,46]. Hence, we suggested that DM can be used as an alternative medicinal fern to replace *Drynaria fortunei* in traditional Chinese material called “Gusuibu” for the treatment of aging-associated symptoms and neurodegenerative disorders. Moreover, we differentiated between DM and *Drynaria fortunei* because the medicinal ferns have different phytoconstituent profiles. This protective mechanism of DM extract against 6-OHDA-induced oxidative damage and apoptosis might be related to its radical scavenging capacity, maintaining the mitochondrial function to inhibit Bcl-2/caspase-3 cascade and activating intracellular antioxidant defenses (including GSH recycling as well as HO-1 and NQO-1) by modulating the activation of the PI3K/AKT/GSK-3β pathway (Figure 8).

## Figures and Tables

**Table 1:** The antioxidant phytoconstituents of aqueous extracts of Davalliaceae species.

Samples	Total Phenolics (mg of catechin/g)	Flavonols (mg of quercetin/g)	Phenylpropanoids (mg of verbascoside/g)	Anthocyanidin (mg of cyanidin/g)
AP	231.26 ± 5.74	13.70 ± 0.20	152.87 ± 3.95	2.06 ± 0.08
DF	141.25 ± 8.65	6.26 ± 0.18	124.37 ± 6.23	1.28 ± 0.10
DG	18.28 ± 1.58	12.24 ± 0.18	14.82 ± 1.56	1.55 ± 0.03
DM	267.02 ± 4.99	11.81 ± 0.26	170.06 ± 9.73	2.67 ± 0.26
DS	172.95 ± 3.17	13.88 ± 0.21	87.41 ± 6.27	0.68 ± 0.03

Data were expressed as mean ± SD (*n* = 3). AP: *Araiostegia perdurans*, DF: *Davallia formosana*, DG: *Davallia griffithiana*, DM: *Davallia mariesii*, DS: *Davallia solida*.

**Table 2:** Free scavenging activities of aqueous extracts of Davalliaceae species.

Samples	DPPH Scavenging (mg of catechin/g)	O_2_^˙^ Scavenging (U of SOD/mg)	H_2_O_2_ Scavenging (μmol of trolox/g)	OH^˙^ Scavenging (mg of quercetin/g)	IC_50_ of Lipid Peroxidation Inhibition (mg/mL)	FRAP (mmol of trolox/g)
AP	391.32 ± 43.40	23.11 ± 2.97	1607.27 ± 19.82	19.66 ± 3.25	21.53 ± 0.95	935.76 ± 17.73
DF	166.80 ± 1.76	11.58 ± 0.70	831.12 ± 50.71	23.47 ± 2.04	126.44 ± 15.30	695.34 ± 22.63
DG	11.84 ± 0.49	1.25 ± 0.07	8.43 ± 0.30	3.74 ± 0.28	156.40 ± 12.88	710.29 ± 15.53
DM	264.38 ± 5.95	18.57 ± 1.79	1730.29 ± 70.28	23.79 ± 4.88	31.90 ± 3.76	1066.46 ± 41.43
DS	211.31 ± 1.29	19.93 ± 0.81	1025.77 ± 17.59	13.30 ± 2.52	38.02 ± 3.01	674.78 ± 25.88

Data were expressed as mean ± SD (*n* = 3). AP: *Araiostegia perdurans,* DF: *Davallia formosana,* DG: *Davallia griffithiana,* DM: *Davallia mariesii,* DS: *Davallia solida*, DPPH: 1,1-diphenyl-2-picryhydrazyl, FRAP: ferric ion reducing antioxidant power, SOD: superoxide dismutase.

**Table 3:** Pearson correlation coefficients (*r*) between parameters describing the contents of total phenolics (TP), flavonols (TF), phenylpropanoids (TPP), and anthocyanidins (TA) and different free radical scavenging activities of Davalliaceae species.

	DPPH	O_2_^˙^	H_2_O_2_	OH^˙^	LPO	FRAP	TP	TF	TPP
O_2_^˙^	0.94 *								
H_2_O_2_	0.92 *	0.91 *							
OH^˙^	0.68	0.64	0.80						
LPO	0.93 *	0.88 *	0.85	0.41					
FRAP	0.63	0.50	0.78	0.54	0.68				
TP	0.89 *	0.91 *	0.996 **	0.81	0.81	0.75			
TF	0.29	0.36	0.21	−0.42	0.62	0.25	0.18		
TPP	0.86	0.80	0.95 *	0.94 *	0.67	0.73	0.95*	−0.11	
TA	0.39	0.19	0.54	0.41	0.44	0.94*	0.51	0.07	0.56

* *p* < 0.05, ** *p* < 0.01. DPPH: 1,1-diphenyl-2-picryhydrazyl, FRAP: ferric ion reducing antioxidant power, LPO: lipid peroxidation.

**Table 4:** Effects of aqueous extract of *Davallia mariesii* (DM, 5–100 μg/mL) on glutathione (GSH) and malondialdehyde (MDA) levels, glutathione peroxidase (GPx) and glutathione reductase (GR) activities in B35 Cells exposed to 50 μM 6-hydroxydopamine (6-OHDA).

Samples	GSH (pmol/mg of protein)	GR (mU/mg of protein)	GPx (mU/mg of protein)	MDA (nmol/mg of protein)
Control	6.61 ± 0.16 **	14.45 ± 0.76 **	157.22 ± 6.19 ***	8.80 ± 0.21**
6-OHDA	2.84 ± 0.12	9.78 ± 0.46	71.79 ± 6.82	15.47 ± 1.70
DM 5 μg/mL + 6-OHDA	2.77 ± 0.08	9.74 ± 0.45	73.89 ± 5.77	12.18 ± 0.14
DM 10 μg/mL + 6-OHDA	3.68 ± 0.12	12.00 ± 0.56	106.95 ± 7.39 **	10.92 ± 1.34
DM 50 μg/mL + 6-OHDA	4.50 ± 0.16 *	12.87 ± 0.50 *	119.57 ± 5.37 **	10.74 ± 0.21 *
DM 100 μg/mL + 6-OHDA	4.68 ± 0.13 *	14.05 ± 0.04 **	149.64 ± 9.54 ***	9.84 ± 0.50 **

B35 cells were treated with DM extract (5–100 μg/mL) 1 h before the addition of 6-OHDA (50 μM). Data are expressed as mean ± SEM (*n* = 4). * *p* < 0.05, ** *p* < 0.01, *** *p* < 0.001 as compared to the 6-OHDA group.
